# Cushioning Performance of Specialized Running Socks for Enhanced Shock Absorption and Reduced Plantar Pressure

**DOI:** 10.3390/ma18132941

**Published:** 2025-06-21

**Authors:** Xia Zhou, Pui-Ling Li, Kit-Lun Yick, Annie Yu

**Affiliations:** School of Fashion and Textiles, The Hong Kong Polytechnic University, Hong Kong, Chinaannie.tw.yu@polyu.edu.hk (A.Y.)

**Keywords:** specialized running socks, cushioning properties, plantar pressure, five-toed socks

## Abstract

Running socks play an important role in alleviating foot impact and preventing foot injuries. Despite the variety of commercial options, their cushioning effectiveness is not well understood. This study examines three different types of running socks made of bio-based and synthetic textiles. Material testing includes compression, tensile, and shock absorption, while wear tests assess plantar loading in 10 adult recreational runners on a treadmill. Results show that specialized running socks offer superior shock absorption compared to regular running socks, largely due to fabric thickness and weight. Socks made of high-performance bio-composite fibers significantly reduced maximum peak pressure and impulse in the great toe (*p* < 0.05) and first metatarsal head (*p* < 0.05) during running. Additionally, ground contact time in the forefoot (*p* < 0.05) area was significantly lower with specialized running socks. Compared to regular running socks, five-toed running socks can reduce the pressure load on the forefoot area. These findings can guide the design of specialized sockwear for better foot protection and improved sports performance.

## 1. Introduction

Running has emerged as a prevalent form of physical activity, widely recognized for its efficacy in enhancing both psychological [1] and physiological [2] well-being. However, with the increase in running duration [3,4] and distance [5,6], the prevalence of foot injuries among amateur runners ranges from approximately 31% to 55%. Among these, mechanical dermal injuries such as friction blisters, abrasions, calluses, and black heels are frequently seen in long-distance, trail, and mountain runners. The impact force generated when the foot touches the ground was an important factor in causing mechanical skin injuries, usually 2–3 times the body weight. Socks are an important part of sportswear, and as the main layer in contact with the foot skin, socks play an important role in reducing sports impact and preventing foot injuries [7,8].

**Figure 1:**
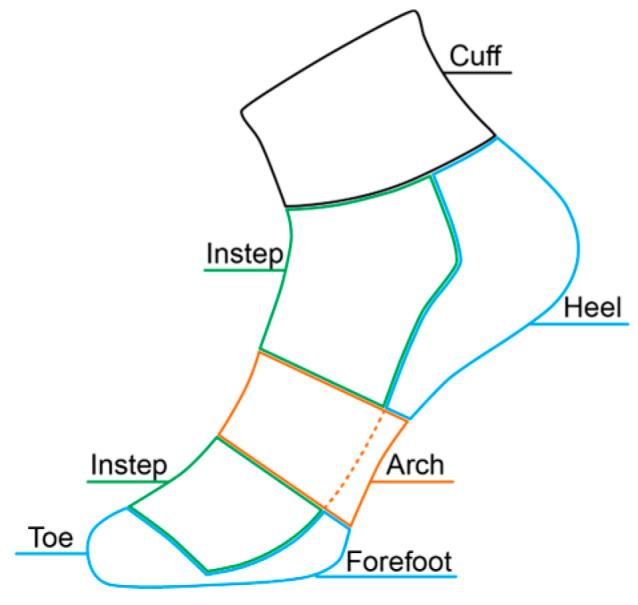
Structure of regular running socks.

Regular running socks are typically constructed from blended natural or synthetic fibers. The cuff of the sock is made of stretch rib knit, the top part of the sock (instep) is made of single jersey, the bottom part of the sock (sole) is made of terry or single jersey, and the toe area is made of seamless knit (Figure 1). However, early studies have found that regular running socks are not effective in foot protection. Cotton socks, even double-layer cotton socks, did not have a significant shock absorption effect during walking compared to barefoot [9]. Regular running socks also did not have a significant effect on vertical or anterior–posterior GRF during walking and running [10]. Therefore, to enhance sports performance and alleviate sports injuries, specialized running socks with biomechanically optimized features are gradually becoming an important part of sports protective equipment.

Currently, specialized running socks are usually composed of special high-performance bio-composite fibers (coolmax^®^, NüWool^TM^, Tencel^®^, etc.), with padded designs in high-pressure areas such as the heel and metatarsal region, and arch support bands woven into the arch region. High-performance bio-composite fibers can improve the performance of fabrics. For instance, bio-ceramic fiber socks have demonstrated superior performance in controlling bacterial colonies and reducing sweating compared to cotton socks [11], and they can also maintain lower foot surface temperatures after physical activities such as running [12]. Additionally, leather-core yarns made from Coolmax^®^/Tencel and PP/Tencel provide high warmth retention, excellent moisture-wicking capabilities, and efficient moisture permeability [13]. Running socks with wool and acrylic cushion soles are superior to barefoot walking in terms of shock absorption [9,10]. Blackmore et al. [10] also showed that specialized running socks containing merino wool significantly reduced the maximum propulsive force during walking. In addition, changing the design of running socks is another way to improve their performance. For example, socks with padding significantly reduced plantar pressure in the bunion region compared to an unpadded control sock. Jiménez-Cano et al. [14] designed a thick pad with cushioning elements in the metatarsal region, which reduced plantar pressure in the central forefoot region by 14.5%. Five-toed socks are another type of specialized running sock that was often mentioned in the prevention of foot injuries in long-distance running [15,16,17]. It is widely believed by those who wear five-toed socks that they may help to increase sensory output to the central nervous system (CNS) by increasing proprioception in the skin of the toes to improve balance [16]. Shinohara and Gribble [18] indicated that the balance of the group with five-toed socks was statistically significantly better than the barefoot group and the group wearing regular socks. Nevertheless, these studies only compare static balance. However, there is limited research on five-toed socks in terms of cushioning. Comparative studies on the effect of different toe designs on cushioning performance are also limited.

Material tests have been used to evaluate the cushioning properties of running sock fabrics, but there has been very limited research on this. The research on sock material performance has usually limited the type of fiber or structure. For example, Baussan et al. [19] compared the shock absorbing properties of cotton socks with six different structures and found that terry fabrics were able to absorb more impact energy than single jersey. Van Amber et al. [20] compared the compression performance of three structures: single knit, semi terry, and terry, and found that energy absorption was strongly influenced by the fabric structure. However, for running socks in the current market, the sock sole is usually made of terry structure, but at present, the performance of the terry structure is limited. And the material test cannot visually reflect the impact mitigation during running; therefore, some researchers have evaluated the cushioning ability of running socks through wear trials. In early studies, Howarth and Rome [9] utilized the treadmill and impact meter to evaluate the cushioning performance of socks and showed that wearing running socks with wool cushioning soles and acrylic cushioning soles mitigated the impact forces during walking compared to being barefoot. Shaikhzadeh et al. [21] measured dynamic plantar pressures in barefoot and in different socks by using The Gaitview^®^ AFA-50 system and found that wearing cross-over and rib-like socks reduced the average plantar pressure in the forefoot region. González et al. [22] utilized the Footscan^®^ platform to assess plantar pressure after a long-distance run. Zhang et al. [23] utilized the Pedar-X in-shoe system to investigate the distribution of plantar pressure in diabetic patients during walking. Current research on the cushioning performance of running socks has predominantly focused on either material testing or wear trials in isolation. However, limited studies have integrated both material testing and human subject evaluations to comprehensively assess the cushioning properties of running socks.

The main objectives of this study were to (1) investigate the effects and differences in cushioning performance of the three types of running socks through fabric testing, and (2) investigate the role of sock design in cushioning performance by comparing the dynamic plantar pressures during running in the three types of running socks.

## 2. Materials and Methods

### 2.1. Material

Eight different commercially available running socks were selected for this study (Table 1). The material specifications of sock samples are given in Table 2. Regular plain running socks made of bio-based and/or synthetic materials are sourced for recreational running activities. Specialized running socks include specialized plain running socks and specialized five-toed running socks, which are blended from high-performance bio-composite fibers, and are also selected for competitive running activities.

### 2.2. Experimental Procedure

The experiment was divided into two steps. The first step was to test the mechanical properties related to the cushioning properties of the sole area of the running socks. The second step was to collect data on plantar pressure distribution during running by wearing three types of running socks with different toe designs.

Before testing, the socks had been conditioned in the standardized environment for 24 h. All tests were carried out in an air-conditioned room under a relative humidity of 60.0 ± 2% and a temperature of 20.0 ± 2 °C.

#### 2.2.1. Material Tests of Sock Sole Regions

The compression properties of fabrics were measured using the KES-FB3-A Compression Tester (Kato Tech Co., Ltd., Tokyo, Japan) in accordance with JIS L 1096-2010 Standard Test for Fabric and Knitted Fabric Testing Method. A sample with dimensions of 5 cm × 20 cm (due to limited sample size) was placed flat in the center of the compression table to avoid deflection. The indenter was gently released to ensure that the initial contact force was close to zero (to avoid pre-compression interference). The compression speed was 0.02 mm/s, the maximum load detection was 50 gf/cm^2^, and each fabric was tested three times.

The tensile and shear characteristics of fabrics were measured using the KES-FB1-A tensile and Shear Tester (Kato Tech Co., Ltd., Tokyo, Japan) in accordance with JIS L 1096-2010 Standard Test Methods for Fabrics and Knitted Fabrics. The sample width was set to 5 cm, the maximum load was 100 gf/cm, and the share angle was 8°. Due to the size limitation of the selected socks, only the toe-to-heel fabric direction was assessed. After the test program was set up, the sample fabric was clamped flat into the fixture for fixation to ensure that there was no slippage or creasing, and was tested three times.

An impact force reduction test was used on a customized test rig [24] to simulate free-fall motion. Prior to the test, the sample was placed on the bottom of the load cell. A ball bearing was released from a 400 mm high platform, and the highest impact force was measured by the load cell. The peak force attenuation ratio (PFAR) is defined as the percentage reduction in the peak force transmitted by the material relative to the peak force in the absence of the cushioning material.

(1) $$PFAR=\left(1-\frac{F_{peak,material}}{F_{peak,\;bear}}\right)\times 100\%$$

where PFAR is the impact force reduction of the sample (%), *Fpeak*, material is the peak force measured for the sample (N), and *Fpeak*, bear is the peak force measured for the load cell surface.

#### 2.2.2. Wear Test

Ten healthy male amateur runners (age: 25.6 ± 2.5 years; body mass: 70.7 ± 6.2 kg; height: 175.5 ± 3.9 cm; shoe size: EU 42–43), all right-leg dominant heel strikers and normal foot with a weekly running distance ≥ 20 km, participated in this study. The participants had no history of neuromuscular, vestibular, or visual disorders, or lower extremity injuries within the past six months. The participants provided informed consent prior to the experiment. The study was approved by the local ethics committee.

Three sock types were tested: regular plain running socks (R2), specialized plain running socks (P2), and specialized five-toed socks (F1). Participants wore uniform footwear (Kinghealth:C-8303) and apparel (DECATHLON: Men’s cooling T-shirt 540 and Men’s Long Swimming Boxers).

In-shoe plantar loads were measured using the Novel Pedar-X system (99 force sensors; 50 Hz sampling rate). First, the participants walked on a treadmill for 5 min at a speed of 7.6 m/s while wearing randomly assigned socks to acclimate to the novel socks. Subsequently, following the Novel Pedar-X system, the trial was initiated, requiring the participants to run on the treadmill at 3.8 m/s. After one minute of running, data collection began with three trials of each pair of socks, each lasting 20 s. A five-minute rest interval was implemented between sets. A minimum of ten complete right-foot plantar pressure data cycles during running were collected for each sock type.

**Figure 2:**
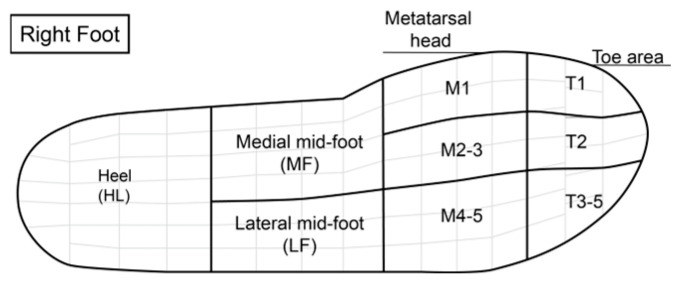
Pedar-X masks.

Plantar loading parameters were analyzed across nine regions (Figure 2), including maximum peak pressure (MPP), contact area (CA), ground contact time (GCT), and pressure time integral (PTI). Mean values from ten successful steps per participant were calculated using the software of Pedar-X Expert SD card version 28.3.8.7.

### 2.3. Data Analysis

All data were presented as mean and standard deviation (SD) and analyzed by IBM^®^ SPSS^®^ Statistics 26. Data was tested for normal distribution using the Shapiro–Wilk test, and homoscedasticity was verified using the Levene test. Material test data were analyzed using the independent samples *t*-tests, then Pearson correlation analysis was performed. The one-way (different running socks) repeated measures analysis of variance (ANOVA) was used for selected plantar loading variables, and post hoc multiple comparisons were performed using the least significant difference (LSD) method. Significance was set at alpha < 0.05.

## 3. Results and Discussion

### 3.1. Material Properties of Sock Sole Regions

#### 3.1.1. Tensile and Shear Properties of Sock Sole Regions

**Figure 3:**
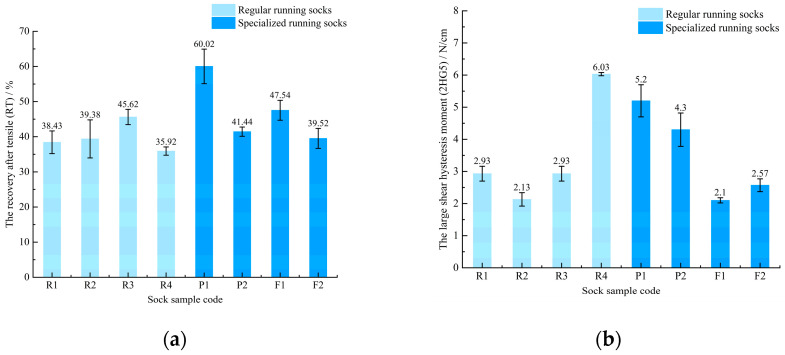
Tensile and shear properties of regular and specialized running socks: (**a**) the recovery after tensile (RT); (**b**) the large shear hysteresis moment (2HG5).

Figure 3 presents the tensile and shear properties measurements of the sock samples. The tensile properties are characterized by the tensile recovery rate (RT), with values approaching 100% reflecting enhanced tensile recovery performance. As shown in Figure 3a, P1 has the highest tensile recovery (RT). Except for P1, the remaining samples show no significant differences in resilience under tensile conditions.

The shear behavior is expressed through the large shear hysteresis moment (2HG5), where higher values correspond to reduced shear deformation recoverability. Shown in Figure 3b, the test results showed that samples R4, P1, and P2 had the worst shear deformation recovery when they received shear force, while the remaining five samples had good recovery performance under shear force.

#### 3.1.2. Compression Properties of Sock Sole Regions

**Figure 4:**
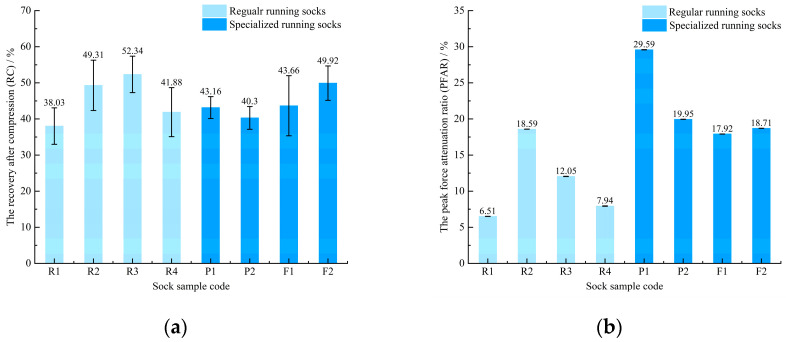
Compression and impact force reduction properties of regular and specialized running socks: (**a**) the recovery after compression (RC); (**b**) the peak force attenuation ratio (PFAR).

Figure 4a presents the compression properties of the sock samples. The compression properties of the fabric are quantified by the recovery rate after compression (RC), where a value closer to 100% indicates superior compression resilience. As illustrated in Figure 4a, R3 exhibited the highest compression recovery, followed by F2. This observation may be attributed to the fiber composition, with R3 comprising polyester yarns and Elastane, while F2 is composed of wool and acrylic fibers. This aligns with previous findings by Cüreklibatır Encan and Marmaralı [25], who reported that terry fabrics containing polyester yarns demonstrate higher compression recovery than those composed of acrylic and cotton fibers.

#### 3.1.3. Impact Force Reduction Properties of Sock Sole Regions

Figure 4b presents the impact force reduction properties of the sock samples. The impact force reduction capacity is assessed using the peak force attenuation ratio (PFAR), with higher PFAR values signifying greater energy absorption capability. As shown in Figure 4b, the peak force reduction ratio of the specialized running socks was generally higher than that of the regular running socks, with the highest reduction ratio of 29.59% for P1.

Notably, the three wool-based socks—R2, P1, and F2—exhibited superior cushioning performance and relatively high compression recovery percentages (over 48%) compared to socks composed of other fibers. This phenomenon may be attributed to the inherent elasticity of wool fibers, which facilitates rebound during fabric deformation. Consequently, higher wool content may further contribute to enhanced peak impact mitigation. Several earlier studies have also confirmed that wool fibers have good elasticity, which improves the cushioning performance of running socks [9,10]. However, these studies mainly focus on pressure.

#### 3.1.4. Relationship Between Sockwear Structural Parameters and Material Properties

As analyzed by the independent samples *t*-test, the differences between the cushioning performance parameters of the regular running socks and the specialized running socks were mainly in the peak force attenuation ratio (t = −2.684, *p* < 0.05), while the differences in compression, tensile, and shear had no statistical significance (Table 3). Overall, specialized running socks reduce peak force by 10% more than regular running socks.

**Figure 5:**
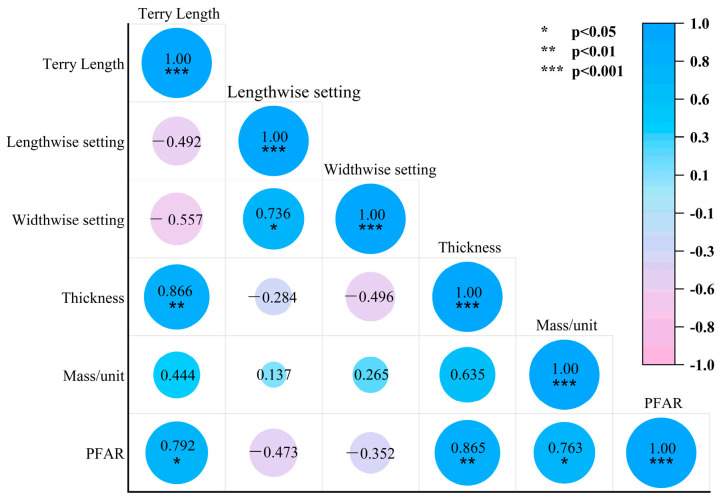
Correlation analysis between basic specification parameters and PFAR.

The peak force reduction ratio was found to be significantly correlated (ρ = 0.865; *p* ≤ 0.001) with fabric thickness, terry length (ρ = 0.792; *p* ≤ 0.05), and fabric mass/unit (ρ = 0.763; *p* < 0.05) by Pearson correlation analysis (Figure 5).

This study investigated the parameters related to the cushioning performance of regular running sock and specialized running sock fabrics. The results showed that the significant difference between the performance of regular running sock and specialized running sock fabrics was in the peak force reduction ratio, and there was a significant strong correlation between the peak force reduction ratio and the fabric thickness. The peak force reduction ratio of running socks ranged from 6.5% to 30%, with the thickest socks, P1 (4.76 mm), demonstrating a peak force reduction ratio of 30%. The terry structure represents one of the primary constructions in the sole design of contemporary running socks. Among the eight socks selected, the terry length in specialized running socks was concentrated within the 2.0–3.0 mm range, generally exceeding that of regular running socks. This design increased the initial fabric thickness, thereby enhancing cushioning capacity. The shape of the terry loop as it is molded did not affect the cushioning properties of the running sock fabric. This was consistent with earlier studies. Prior studies have established that terry structures enhance cushioning compared to single jersey knit [19,26]. Van Amber et al. [20] found that the differences in the compressive behavior of the three fabrics, single jersey, half-terry, and terry, appeared to be related primarily to the initial fabric thickness rather than to inherent structural differences. The terry fabric absorbed the most energy with the thickest thickness. It was also found that the medium-thickness socks were able to reduce peak plantar pressures by more than 15% [27,28,29]. Blackmore et al. [30] found statistically significant correlations between sock thickness and dynamic impact parameters such as peak impact force, time to peak impact force, and loading rate, with reductions ranging from 3% to 20%. This functional similarity was observed in padded and multilayer socks, both achieving load reduction through enhanced thickness. Overall, specialized running socks perform better than regular running socks in terms of cushioning, compression, and tension.

### 3.2. Wear Test on Plantar Pressure Distribution

Table 4 shows the mean maximum peak pressure, pressure time integral, and ground contact time while wearing the three running socks. Compared with wearing regular plain running socks (R2), the specialized running socks (P2 and F1) were able to significantly reduce the mean maximum plantar pressures at T1 (F = 8.313; *p* = 0.009) and M1 (F = 6.322; *p* = 0.014) during running, and significantly reduce the pressure time integrals in the regions of T1 (F = 6.256; *p* = 0.017) and T3–5 (F = 4.052; *p* = 0.029).

Running in regular plain socks (R2) revealed longer ground contact time in the toes, metatarsal [T1 (F =35.559; *p* = 0.000), T2 (F = 6.125; *p*= 0.012) T3–5 (F = 17.064; *p* = 0.000), M1 (F = 13.828; *p* = 0.002), M2–3 (F = 13.416; *p* = 0.012), and M4–5 (F = 8.075; *p* = 0.014)], and total foot (F = 16.234; *p* = 0.000) than running in the specialized running socks (P2 and F1). There were no significant differences between the specialized five-toed running socks (F1) and specialized plain running socks (P2) in terms of mean maximum peak pressure, pressure time integral, or ground contact time. No significant difference in contact area was found between the three socks in the full foot and all foot areas.

In this study, the data of the dynamics mean peak plantar pressure, pressure time integral, and ground contact time (from heel strike to forefoot off the ground) in amateur runners wearing three types of running socks were analyzed. To minimize the effect of fabric cushioning properties on structure and fiber, three socks (R2, P2, and F1) with similar fabric thickness, terry length, mass/unit of the fabric, and cushioning properties, but with different toe designs, were selected for the experiment for wear trails. The primary findings indicate that specialized running socks (P2 and F1) were effective in reducing pressure loads and running impulse in the foot with the big toe and first metatarsal head compared to regular running socks (R2), which indicated that specialized running socks have the potential to mitigate the risk of mechanical stresses and associated injuries. This was consistent with the results of previous studies [10,30]. Despite the similar thickness of the three running socks, the plantar loading in the metatarsal and heel areas of the specialized running socks (P2 and F1) remained lower than that of the regular running sock (R2). This may be due to the high-performance bio-composite fibers (e.g., Drynamix^®^, mohair, Lycra^®^, and Drymax^®^) incorporated in the specialized running socks, which have excellent elasticity and recovery properties [9,31], and continue to show good elasticity and compression resistance during high impact.

In the toe region, specialized five-toed running socks (F1) significantly reduced pressure peaks in the high-load forefoot area compared to regular running socks (R2). And the plantar pressure distribution of specialized five-toed running socks (F1) was comparable to that of specialized plain running socks (P2). However, a notable reduction in thickness was observed in the toe region of five-toed socks (F1) relative to plain running socks (R2 and P2). This difference arises because the split-toe design of five-toed running socks (F1) utilizes a single jersey knit, whereas plain running socks (R2 and P2) employ terry structure. Terry fabric exhibited higher thickness than single jersey knit fabric. The current findings indicate that despite reduced thickness in the split-toe region, five-toe socks effectively mitigated plantar loading. This study thus validates the potential of five-toe sock designs for load reduction. Although research on pressure distribution in commercially available running socks remains limited, these results propose a novel design paradigm: optimizing sock performance through structural modifications—rather than solely relying on thickness augmentation—can enhance cushioning efficacy.

There are some limitations to this study. Although several previous studies have found that running on a treadmill reduces forefoot plantar strength and pressure, some researchers believe that evaluating the running kinematics of footwear on a treadmill may lead to non-generalizable conclusions [32]. In addition, the impact of running socks may also be influenced by footwear, so it is necessary to conduct in-depth measurements of foot movement in a barefoot state or control for the consistency of footwear’s initial impact on the foot. Furthermore, this study only examined the initial characteristics of commercially available socks. In future research, it would be worthwhile to further investigate the durability and functional characteristics of running socks after multiple washes to gain a deeper understanding of their functionality during actual physical activity.

## 4. Conclusions

This study revealed that differences in cushioning performance between regular and specialized running socks, as evidenced through material testing, were primarily manifested in peak force attenuation ratio directly correlated with fabric thickness. Specialized running socks have a 10% higher peak force attenuation ratio than regular running socks. Furthermore, specialized running socks demonstrated reduced average peak pressure at the great toe and first metatarsal regions, along with shorter forefoot ground contact duration during running, compared to regular running socks. These effects were attributed to sock toe design variations and fiber material characteristics. Additionally, the five-toed sock design exhibited potential to mitigate plantar loading during running. Professional running socks consistently display enhanced cushioning performance under both impact testing and plantar pressure loading conditions. This study enhances the comprehension of cushioning performance in commercially available specialized running socks and offers novel insights for optimizing running socks, thus promoting the development of foot sports health.

## Figures and Tables

**Table 1:** Specification of tested running socks.

Code	Fiber Content	Pattern Structure	Wearing Features (By Producer)	Picture
Regular plain running socks				
R1	67.9% Cotton, 27.7% Polyester, 4.4% Elastane	Welt: double-sided ribbing; Instep: single jersey; Arch part: single jersey with inlaid rubber thread; Heel and toe: terry knitted	Stretchy and comfortable	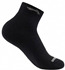
R2	52.1% Wool, 30.7% Polyamide, 13.2% Polyester, 4% Elastane	Welt: double-sided ribbing; Instep: single jersey; Arch part: terry knitted with inlaid rubber thread (3:1); Heel and toe: terry knitted	Soft, good resilience	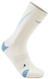
R3	89% Polyester, 11% Elastane	Welt: double-sided ribbing; Instep: single jersey; Arch part: terry knitted with inlaid rubber thread (2:1); Heel and toe: terry knitted	Anti-bacterial, anti-odor, breathable	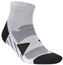
R4	94% Nylon, 6% Spandex	Welt: double-sided ribbing; Instep: single jersey; Arch part: single jersey with inlaid rubber thread (2:1); Heel and toe: terry knitted	Moisture wicking, anti-odor	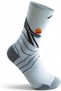
Specialized running socks (Two plain running socks and two five-toed running socks)				
P1	28% Drynamix^®^, 27% Mohair 43% Nylon, 2% Elastane	Welt: double-sided ribbing; Instep: single jersey; Arch part: terry knitted with inlaid rubber thread (2:1); Heel and toe: terry knitted	Anatomically shaped for L/R, V-Tech arch support design	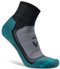
P2	52% Drymax^®^ Olefin, 32% Polyester, 11% Elastane, 5% Nylon	Welt: double-sided ribbing; Instep: single jersey; Arch part: terry knitted with inlaid rubber thread (3:1); Heel and toe: terry knitted	With Drymax dual-layer active wicking material	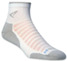
F1	39% Coolmax^®^, 58% Nylon, 3%Lycra^®^	Welt: double-sided ribbing; Instep: single jersey; Arch part: terry knitted with inlaid rubber thread (2:1); Heel: terry knitted; Toe: single jersey	Five-toe design prevents blisters and redness	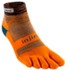
F2	43% NüWoolTM, 43% Acrylic 12% Nylon, 2% Lycra^®^	Welt: double-sided ribbing; Instep: single jersey; Arch part: terry knitted with inlaid rubber thread (2:1); Heel: terry knitted; Toe: single jersey	Five-toe design, moisture management	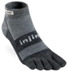

**Table 2:** The basic specification of sock samples (heel and forefoot areas of the running socks, excluding the toe of five-toed socks).

Code	Terry Length (mm)		Thickness (mm)		Lengthwise Setting (cm^−1^)		Widthwise Setting (cm^−1^)		Mass/Unit (g/m^2^)		Front Organization	Terry Structure
	Mean	S.D.	Mean	S.D.	Mean	S.D.	Mean	S.D.	Mean	S.D.		
R1	0.85	0.05	1.95	0.04	13.83	0.14	11.14	0.13	371.29	19.67	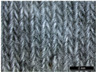	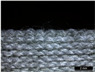
R2	2.27	0.07	3.98	0.02	12.25	0.21	8.94	0.10	630.45	4.14	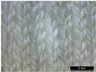	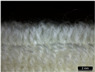
R3	1.24	0.07	2.70	0.07	14.68	0.14	8.32	0.07	419.07	23.69	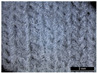	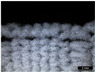
R4	1.50	0.08	2.74	0.13	13.35	0.24	11.70	0.06	747.17	10.57	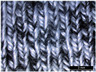	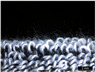
P1	2.35	0.07	4.76	0.09	14.50	0.18	10.48	0.12	1322.09	28.48	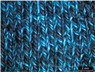	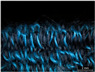
P2	2.92	0.08	3.36	0.10	7.77	0.08	7.43	0.22	535.75	14.16	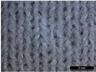	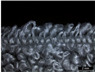
F1	2.02	0.10	3.61	0.14	13.15	0.12	9.50	0.26	724.83	31.70	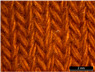	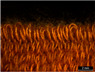
F2	2.04	0.10	3.80	0.04	11.63	0.19	8.94	0.06	841.35	0.17	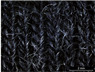	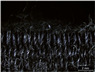

**Table 3:** Independent samples *t*-test for selected properties means.

Dependent Variable	Mean (S.D.)		t	*p*
	Regular Running Sock	Specialized Running Sock		
PFAR (%)	11.27 (5.51)	21.54 (5.43)	−2.684	0.036 *
RC (%)	45.39 (6.59)	46.01 (7.43)	−0.125	0.904
RT (%)	42.46 (2.87)	47.00 (8.91)	−0.969	0.370
2HG5 (N/cm)	2.59 (0.37)	3.47 (1.43)	−1.198	0.276

* Significant differences at the 0.05 level and bold.

**Table 4:** Comparison of insole loading parameters for running on different socks.

Variable	Mean (Standard Deviation)			
	Regular Plain Running Sock (R2)	Specialized Five-Toed Running Sock (P2)	Specialized Plain Running Sock (F1)	*p*
**Maximum peak pressure (kPa)**				
T1	222.3 (55.1)	198 (53.5)	199.6 (56.1)	0.009 *
T2	150.1 (23.7)	153.9 (31.1)	153 (25.4)	0.854
T3–5	116.2 (22.6)	109.4 (26.1)	113.9 (26.4)	0.426
M1	377.2 (88.7)	349.5 (93.9)	349.2 (98.1)	0.014 *
M2–3	282.8 (43.6)	280.7 (53.1)	279.9 (55.4)	0.822
M4–5	200.7 (28.5)	206.9 (35.0)	200.7 (27.5)	0.501
MF	83.8 (20.3)	88.6 (18.6)	88.2 (15.8)	0.059
LF	104.5 (19.4)	105.9 (18.3)	103.9 (14.6)	0.725
HL	213.4 (75.2)	205.1 (58.1)	210.9 (45.9)	0.717
Total	385.8 (77.4)	358.2 (79.5)	355.3 (91.9)	0.011 *
**Pressure time integral (kPa**∗**s)**				
T1	28.1 (5.2)	25 (6.2)	26.2 (6.1)	0.017 *
T2	21.3 (4.3)	21.3 (4.9)	21.7 (4.0)	0.878
T3–5	18.1 (4.5)	16.1 (4.6)	17.4 (4.8)	0.039 *
M1	47.8 (11.3)	45.4 (12.5)	45.5 (12.6)	0.103
M2–3	38.4 (6.7)	38.2 (8.2)	38.4 (8.4)	0.945
M4–5	29.2 (6.0)	30 (7.1)	29.3 (6.2)	0.636
MF	8.3 (2.5)	8.8 (2.4)	8.6 (2.3)	0.059
LF	13 (3.3)	13.1 (3.0)	12.9 (2.5)	0.856
HL	18.1 (5.7)	20.3 (7.5)	20.4 (10.5)	0.520
Total	57.7 (9.2)	55.5 (9.7)	55.5 (11.6)	0.085
**Ground contact stance (ms)**				
T1	214.2 (12.8)	197.2 (14.4)	200.2 (13.6)	0.000 *
T2	214.5 (18.4)	204 (19.8)	203.6 (14.8)	0.012 *
T3–5	220.8 (20.1)	205.4 (18.4)	205.6 (18.1)	0.000 *
M1	223.9 (18.7)	211.4 (21.7)	208.2 (18.4)	0.002 *
M2–3	226.6 (15.6)	215.6 (18.4)	213 (15.6)	0.002 *
M4–5	239 (18.8)	225 (18.2)	224.6 (17.3)	0.014 *
MF	132.4 (14.1)	130.4 (16)	131.1 (16.6)	0.689
LF	189.1 (37.4)	170.2 (28.7)	171.6 (21.4)	0.051
HL	158.8 (36.9)	161.4 (43.5)	159 (43.9)	0.961
Total	247.5 (12.2)	233.2 (15.7)	235.4 (13.4)	0.000 *

* Significant differences at the 0.05 level and bold.

## Data Availability

The original contributions presented in this study are included in the article. Further inquiries can be directed to the corresponding author.
